# Radiomics Analysis Based on Magnetic Resonance Imaging for Preoperative Overall Survival Prediction in Isocitrate Dehydrogenase Wild-Type Glioblastoma

**DOI:** 10.3389/fnins.2021.791776

**Published:** 2022-01-28

**Authors:** Shouchao Wang, Feng Xiao, Wenbo Sun, Chao Yang, Chao Ma, Yong Huang, Dan Xu, Lanqing Li, Jun Chen, Huan Li, Haibo Xu

**Affiliations:** ^1^Department of Radiology, Zhongnan Hospital of Wuhan University, Wuhan, China; ^2^Department of Neurosurgery, Zhongnan Hospital of Wuhan University, Wuhan, China; ^3^Department of Radiation and Medical Oncology, Zhongnan Hospital of Wuhan University, Wuhan, China; ^4^Precision Health Institute, GE Healthcare, Shanghai, China

**Keywords:** radiomics, isocitrate dehydrogenase wildtype, glioblastoma, MRI, nomogram

## Abstract

**Purpose:**

This study aimed to develop a radiomics signature for the preoperative prognosis prediction of isocitrate dehydrogenase (IDH)-wild-type glioblastoma (GBM) patients and to provide personalized assistance in the clinical decision-making for different patients.

**Materials and Methods:**

A total of 142 IDH-wild-type GBM patients classified using the new classification criteria of WHO 2021 from two centers were included in the study and randomly divided into a training set and a test set. Firstly, their clinical characteristics were screened using univariate Cox regression. Then, the radiomics features were extracted from the tumor and peritumoral edema areas on their contrast-enhanced T1-weighted image (CE-T1WI), T2-weighted image (T2WI), and T2-weighted fluid-attenuated inversion recovery (T2-FLAIR) magnetic resonance imaging (MRI) images. Subsequently, inter- and intra-class correlation coefficient (ICC) analysis, Spearman’s correlation analysis, univariate Cox, and the least absolute shrinkage and selection operator (LASSO) Cox regression were used step by step for feature selection and the construction of a radiomics signature. The combined model was established by integrating the selected clinical factors. Kaplan–Meier analysis was performed for the validation of the discrimination ability of the model, and the C-index was used to evaluate consistency in the prediction. Finally, a Radiomics + Clinical nomogram was generated for personalized prognosis analysis and then validated using the calibration curve.

**Results:**

Analysis of the clinical characteristics resulted in the screening of four risk factors. The combination of ICC, Spearman’s correlation, and univariate and LASSO Cox resulted in the selection of eight radiomics features, which made up the radiomics signature. Both the radiomics and combined models can significantly stratify high- and low-risk patients (*p* < 0.001 and *p* < 0.05 for the training and test sets, respectively) and obtained good prediction consistency (C-index = 0.74–0.86). The calibration plots exhibited good agreement in both 1- and 2-year survival between the prediction of the model and the actual observation.

**Conclusion:**

Radiomics is an independent preoperative non-invasive prognostic tool for patients who were newly classified as having IDH-wild-type GBM. The constructed nomogram, which combined radiomics features with clinical factors, can predict the overall survival (OS) of IDH-wild-type GBM patients and could be a new supplement to treatment guidelines.

## Introduction

Among all primary brain and other central nervous system tumors, gliomas account for about 26% (Ostrom et al., 2018). Among all malignant brain and other central nervous system tumors, glioblastoma (GBM) is the most common (Soomro et al., 2017). Despite the advances in surgery and chemoradiotherapy being great, the prognosis of GBM patients is still poor, with a median survival of only 12–14 months (Henson et al., 2005; Stupp et al., 2005; Van Meir et al., 2010; Lu et al., 2020; Soltani et al., 2021). In addition, several previous studies have confirmed that the status of isocitrate dehydrogenase (IDH) mutation has a great impact on the prognosis of GBM patients (Parsons et al., 2008; Hartmann et al., 2013; Reifenberger et al., 2014). According to the latest classification criteria of the World Health Organization (WHO) in 2021, the common diffuse gliomas of adults have been divided into only three types: astrocytoma, IDH-mutant; oligodendroglioma, IDH-mutant and 1p/19q-codeleted; and glioblastoma, IDH-wild type. IDH-wild-type diffuse astrocytic (NB: diffuse and astrocytic) tumors in adults, if microvascular proliferation or necrosis or telomerase reverse transcriptase (TERT) promoter mutation or epidermal growth factor receptor (*EGFR*) gene amplification or +7/-10 chromosome copy number changes are present, should be diagnosed as IDH-wild-type GBM. Based on the new classification criteria, a more reasonable and personalized prognosis analysis method can be constructed, which will directly affect the condition assessment, targeted treatment, and follow-up management of IDH-wild-type GBM patients (Louis et al., 2021).

In current clinical practice, several factors are usually combined to predict the prognosis of GBM patients, including age, gender, Karnofsky performance status (KPS; Buckner, 2003; Chambless et al., 2015), tumor location, and laterality (Field et al., 2014; Gately et al., 2018), molecular spectrum analysis (Binabaj et al., 2018; Schaff et al., 2020), and treatment plan (Stupp et al., 2009; Herrlinger et al., 2019), among others. In addition, it has been reported that the prognosis of malignant glioma may be affected by some basic characteristics of preoperative MRI, such as the extent of peritumoral edema (PTE), tumor crossing the midline, necrosis, enhancement, and the size of the cyst (Maldaun et al., 2004; Pope et al., 2005; Wu et al., 2015; Hansen et al., 2018). MRI is a non-invasive preoperative routine examination for GBM with the advantage of multi-parameter imaging and excellent soft tissue resolution, which can provide comprehensive structural and functional information of the tumor (Henson et al., 2005; Diehn et al., 2008). Currently, it has been proven that the MRI technology exhibits great potential in predicting the survival of patients with GBM (Gutman et al., 2013; Gevaert et al., 2014; Rao et al., 2016).

Radiomics transforms the subjective and semi-quantitative description of traditional imaging diagnosis (such as the enhancement degree, edema range, and space occupying effect) into objective and quantitative parameters (such as histogram features, and texture features) (Lambin et al., 2012; Peeken et al., 2017; Rogers et al., 2020). Compared to traditional imaging features, these quantitative radiomics features contain a number of invisible tumor biological information, such as tumor heterogeneity, oncogenic processes, invasion, and metastasis (Gillies et al., 2016; Limkin et al., 2017; Peeken et al., 2017; Liu et al., 2019). Therefore, a more effective and reliable method may be to analyze the prognosis of IDH-wild-type GBM by using a comprehensive multivariate model based on radiomics features and clinical risk factors.

This study aimed to develop machine learning models that can be used to predict the prognosis of IDH-wild-type GBM based on the radiomics features extracted from multicenter, multi-parameter MRI images. By constructing a combined Radiomics + Clinical nomogram, the overall survival of IDH-wild-type GBM patients was analyzed and predicted individually.

## Materials and Methods

### Patients

Recruitment of the patients included in this study consisted of two parts. The first part was from our hospital (Zhongnan Hospital of Wuhan University), which was approved by the Medical Ethics Committee of the hospital (approval no. 2020181). Informed consent was waived because of the retrospective nature of the study. Using the Picture Archiving and Communications System (PACS), we searched and enrolled the patients admitted to hospital between June 2016 and December 2020. The second part was from the public databases The Cancer Genome Atlas (TCGA)/The Cancer Imaging Archive (TCIA; Clark et al., 2013). The patient identifiers in the databases were erased and approval of the Institutional Review Committee was not required. All patients were included and selected based on the following exclusion criteria: (1) patients without any sequence of contrast-enhanced T1-weighted image (CE-T1WI), T2-weighted image (T2WI), or T2-weighted fluid-attenuated inversion recovery (T2-FLAIR) images; (2) patients who had received biopsy or operation; and (3) patients with large image artifacts and poor quality images, which may cause deviation in the follow-up process. Finally, a total of 142 patients from multiple centers were included in this study, among which 70 were from our hospital and 72 were from public databases. The detailed patient screening and grouping process is shown in Supplementary Figure 1, while the individual characteristics of these patients are shown in Table 1.

**TABLE 1:** Clinical and traditional imaging characteristics of patients included in the study.

Characteristics	All subjects (*n* = 142)	Training set (*n* = 100)	Test set (*n* = 42)	*p-*value
**Clinical**				
Age (years), mean ± SD	58.72 ± 11.74	58.28 ± 10.86	59.76 ± 13.69	0.535
Gender, *n* (%)				0.870
Male	86 (60.56)	61 (61)	25 (59.52)	
Female	56 (39.44)	39 (39)	17 (40.48)	
KPS, median (range)	80 (40–100)	80 (40–100)	80 (40–100)	
Treatment, *n* (%)				0.802
Standard	120 (84.51)	85 (85)	35 (83.33)	
Non-standard	22 (15.49)	15 (15)	7 (16.67)	
**Traditional imaging**				
Location, *n* (%)				0.061
Frontal	61 (42.96)	45 (45)	16 (38.10)	
Temporal	39 (27.46)	27 (27)	12 (28.57)	
Parietal	23 (16.20)	11 (11)	12 (28.57)	
Occipital	5 (3.52)	5 (5)	0 (0)	
Others	14 (9.86)	12 (12)	2 (4.76)	
Number, *n* (%)				0.741
Single	109 (76.76)	76 (76)	33 (78.57)	
Multiple	33 (23.24)	24 (24)	9 (21.43)	
Tumor crossing the midline, *n* (%)				0.582
Yes	17 (11.97)	11 (11)	6 (14.29)	
No	125 (88.03)	89 (89)	36 (85.71)	
Maximum tumor diameter, mean ± SD	48.67 ± 16.33	49.77 ± 16.79	46.08 ± 15.05	0.202
Maximum edema diameter, mean ± SD	18.46 ± 10.55	18.99 ± 10.24	17.19 ± 10.28	0.377
PTE, *n* (%)				0.465
Minor, <1cm	38 (26.76)	25 (25)	13 (30.95)	
Major, ≥1cm	104 (73.24)	75 (75)	29 (69.05)	
Edema shape, *n* (%)				0.513
Rounded	65 (45.77)	44 (44)	21 (50.00)	
Irregular	77 (54.23)	56 (56)	21 (50.00)	
Edema diameter/tumor diameter, mean ± SD	0.43 ± 0.33	0.44 ± 0.36	0.39 ± 0.24	0.274
Necrosis, *n* (%)				0.744
No	14 (9.86)	9 (9)	5 (11.90)	
Mild	61 (42.96)	42 (42)	19 (45.24)	
Severe	67 (47.18)	49 (49)	18 (42.86)	
Cyst, *n* (%)				0.700
No	83 (58.45)	57 (57)	26 (61.90)	
Small	30 (21.13)	23 (23)	7 (16.67)	
Large	29 (20.42)	20 (20)	9 (21.43)	
Enhancement, *n* (%)				0.815
Not marked	63 (44.37)	45 (45)	18 (42.86)	
Marked	79 (55.63)	55 (55)	24 (57.14)	
OS, median (range)	306 (17–1,185)	296 (23–1,185)	322 (17–1,143)	

*OS, overall survival.*

### Follow-Up

The overall survival (OS) of the patients from our hospital was calculated from the date of surgery to the last follow-up or death, and the preoperative image date was not long before the operation date. The minimum follow-up period to ascertain OS was 18 months after the date of surgery, if patients were still alive. The OS of the patients from TCGA/TCIA databases was obtained from support documents of the TCGA GBM Project.

### Data Flowchart

**FIGURE 1:**
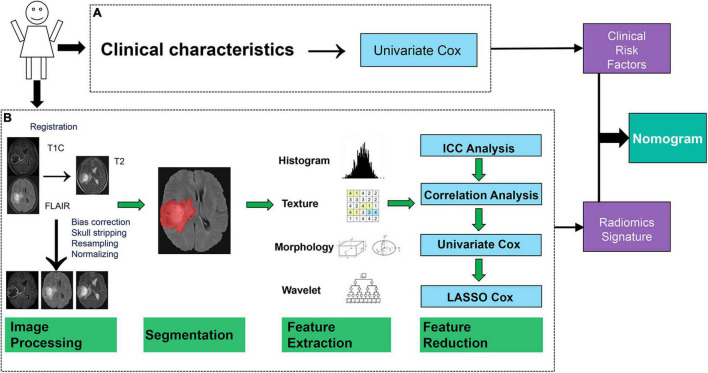
Data flowchart of the study. **(A)** Module of the clinical characteristic analysis. **(B)** Module of the radiomics analysis.

As seen in Figure 1, the data processing of this study consisted of two modules: clinical characteristics and radiomics analysis modules. Clinical risk factors were screened one by one using univariate Cox regression, while radiomics analysis could be divided into five parts: image preprocessing, image segmentation, feature extraction, feature reduction, and step-by-9step model construction. The radiomics and combined (Radiomics + Clinical) models were constructed separately and then compared. Finally, the nomogram was generated as an effective tool to personally predict the 1- and 2-year survival of patients based on the model with better predictive performance.

As shown in Table 1 and Supplementary Figure 1, all 70 samples from our hospital and 30 samples randomly selected from TCGA/TCIA made up the training set (a total of 100 cases), while the remaining 42 samples from TCGA/TCIA made up the test set. All feature analyses and modeling (clinical and radiomics) were operated on the training set and then validated on both the training and test sets.

### Images Acquisition

All preoperative MRI images in our hospital were obtained with a 3.0-T scanner [Umr790 (Philips, Best, Netherlands) or MAGNETOM Trio (Siemens, Erlangen, Germany)] using an 8-channel array coil. The detailed acquisition parameters are summarized in Supplementary Methods.

### Analysis of the Clinical Characteristics

Two radiologists reviewed the images with the double-blind method and negotiated the results together. Referring to a previously reported method (Wu et al., 2015), we collected radiological characteristics such as qualitative indicators (location, number, tumor crossing the midline, degree of edema, edema shape, degree of necrosis, degree of cystic change, and degree of enhancement) and quantitative indicators (maximum tumor diameter, maximum edema diameter, and maximum edema diameter/maximum tumor diameter). In addition to the above, information on the age, gender, KPS, and treatments were included in this study. Univariate Cox analysis was performed between OS and the clinical characteristics individually to screen the clinical risk factors.

### Image Analysis

Before image analysis, T2-FLAIR images and CE-T1WI were strictly registered to T2WI using MATLAB software with SPM. Then, the CE-T1WI, T2WI, and the T2-FLAIR images were preprocessed using Analysis Kit software (GE Healthcare, Chicago, IL, United States). This included bias correction, skull stripping, and image resampling and normalization. One radiologist (SW) used ITK-SNAP to manually segment the regions of interest (ROIs). The region with a high signal on the T2-FLAIR image was drawn as the ROI.

For each MRI sequence (CE-T1WI, T2WI, and T2-FLAIR), 402 radiomics features were extracted for the ROI using AK Software (see Supplementary Methods). Then, all 1,206 features were combined for further modeling.

Twenty samples were randomly selected from all samples to test feature reliability and reproducibility. Feature extraction by two authors (SW and WS) was initially analyzed. The procedures were completed together to guarantee the productivity and stability of the features (see Supplementary Methods).

### Feature Reduction and Radiomics Signature Construction

Four methods were used in the feature reduction and modeling processes (Figure 1B). Firstly, inter- and intra-class correlation coefficients (ICCs) were calculated to assess the stability and repeatability of each radiomics feature. Features with ICCs larger than 0.8 were considered to be robust and were retained for further analysis (see Supplementary Methods). Thereafter, Spearman’s correlation coefficient for each pair of features was calculated. Two features with a correlation coefficient (*r*) larger than 0.9 meant that there is a high correlation between them, and one of these features was erased randomly.

Subsequently, univariate Cox analysis was performed on each radiomics feature to evaluate the ability of a single feature to predict OS. Features with *p*-values less than 0.05 were considered to be statistically significant and were retained in the least absolute shrinkage and selection operator (LASSO) Cox model for further analysis.

Least absolute shrinkage and selection operator Cox is a multivariate Cox method with L1 regularization. During modeling, the number of features could be reduced by imposing a penalty term to the feature weights. In this work, the key penalty coefficient of the LASSO Cox model was determined by the use of fivefold cross-validation.

The radiomics signature was established by a linear combination of the retained features (non-zero coefficients) after LASSO Cox analysis. Kaplan–Meier (KM) survival curve analysis of OS based on the optimal cutoff value of the constructed radiomics and combined models was performed to stratify patients into high-risk and low-risk groups in the training set and test set, respectively. The optimal cutoff value of the radiomics signature was determined through traversing all values of the radiomics signature to obtain the best stratification result. The log-rank test was used for comparisons of the differences between the high- and low-risk patients stratified using the constructed models. Ultimately, the Radiomics + Clinical model was constructed using multivariate Cox analysis by combining the clinical risk factors and the radiomics signature. Proportionality assumption was tested based on the Schoenfeld residuals test. The consistency of model prediction was assessed using the C-index.

### Nomogram Construction

The nomogram transformed the corresponding model into a simple and visual graph, making the results of the prediction model more distinct and of higher use value (see Supplementary Methods). Calibration curves were used to assess the consistency between the actual observation outcome and the nomogram prediction.

### Statistical Analysis

The distributions of the clinical characteristics in the training and test sets were represented in the form of mean ± standard deviation or proportion according to the variable type. Group differences for these variables were compared using an independent samples *t*-test, chi-square test, or the Mann–Whitney U test according to the variable type and distribution.

Statistical analysis was performed using R software (version 3.6.3).^1^ The following R packages were used: “glmnet” to perform the LASSO Cox regression analysis, the “survival” and “survminer” packages to implement the Kaplan–Meier analysis, and the “rms” package to implement the nomogram construction and calibration evaluation. A two-sided *p*-value < 0.05 was considered to be statistically significant for all statistical analyses.

## Results

### Clinical Characteristics and Overall Survival

As presented in Table 1, no significant difference in the OS and clinical characteristics was found between the training and test sets. After univariate Cox analysis (Table 2), four independent risk factors, which included age, KPS, tumor crossing the midline and maximum tumor diameter significantly influenced the OS.

**TABLE 2:** Univariate analysis of the clinical and traditional imaging factors with the overall survival (OS) of patients using Cox regression model.

Factors	HR	95% CI	*p*-value
Age	1.024	1.001–1.048	0.038*
Gender	1.064	0.697–1.623	0.774
KPS	0.987	0.976–0.998	0.021*
Treatment	0.708	0.442–1.134	0.151
Location	0.749	0.326–1.191	0.277
Number	1.18	0.778–1.788	0.436
Tumor crossing the midline	2.417	1.42–4.11	0.001*
Maximum tumor diameter	1.017	1.005–1.029	0.007*
Maximum edema diameter	0.996	0.979–1.013	0.626
PTE	0.896	0.604–1.327	0.583
Edema shape	1.069	0.753–1.518	0.708
Edema/tumor diameter	0.706	0.397–1.255	0.236
Necrosis	0.961	0.613–1.740	0.897
Cyst	1.108	0.717–1.484	0.815
Enhancement	0.942	0.663–1.337	0.737

PTE, peritumoral edema. *p < 0.05.

### Image Analysis and Radiomics Signature Construction

**FIGURE 2:**
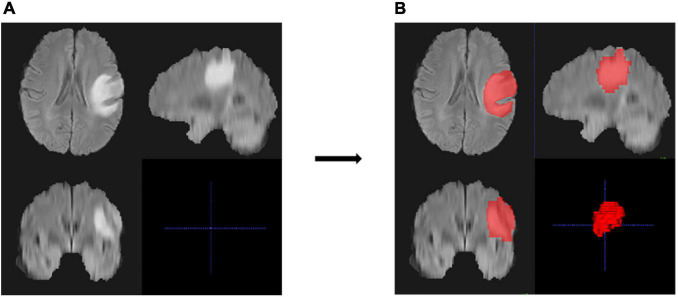
An example of region of interest (ROI) and the segmentation results of the MRI images of one patient. **(A,B)** Cross-section views of images before segmentation **(A)** and after manual segmentation **(B)**. The lesion in the original image was high and showed a bright signal **(A)**, and the ROI of the lesion area is represented as red after segmentation **(B)**.

**FIGURE 3:**
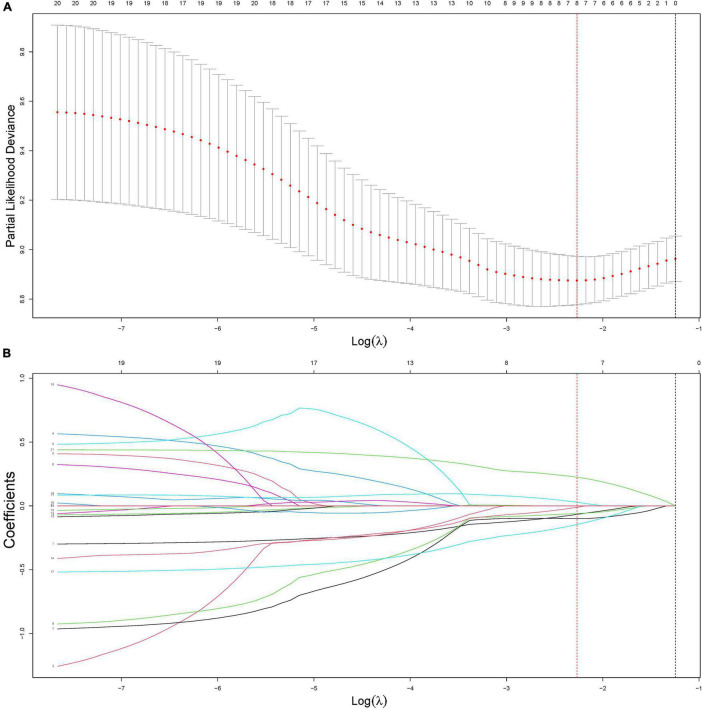
Construction of the radiomics signature using least absolute shrinkage and selection operator (LASSO) Cox regression. **(A)** An optimal tuning parameter (λ) in the LASSO regression model was selected using fivefold cross-validation and the partial likelihood deviance rule. Two *vertical dashed lines* were drawn for two criteria: (1) the minimum of the partial likelihood deviance (lambda.min, *red dashed*) and the least feature number in the range of 1 standard error around lambda.min (lambda.1SE, *black dashed*). In this study, lambda.min (0.1035244) was selected to minimize the partial likelihood deviance. **(B)** LASSO coefficient profiles of the features. According to the fivefold cross-validation in panel **(A)**, the optimal λ value was determined at lambda.min, and the corresponding features with non-zero coefficients were included in the construction of the radiomics signature.

Figure 2 shows an example of the lesion area segmented as ROI using the manual segmentation method. For the ROI in each MRI sequence image of a patient, 402 features were extracted. Thus, a total of 1,206 quantitative features were obtained for one patient, of which 614 features with ICC > 0.8 were retained for further analysis. Spearman’s correlation analysis reduced the features to 241, and then by univariate Cox analysis to 45. Figure 3 shows the results of feature reduction using LASSO Cox. Ultimately, 8 features were retained (Table 3), which made up the radiomics signature through linear combination.

**TABLE 3:** Features and their corresponding coefficients in the radiomics signature.

	Feature category	Feature name	Coefficients
Radiomics	Contrast	C_ShortRunEmphasis_ AllDirection_offset4_SD	–0.102
	T2	T2_ClusterShade_AllDirection_ offset1_SD	–0.099
		T2_ClusterShade_AllDirection_ offset7_SD	–0.073
		T2_Correlation_angle45_ offset7	0.059
		T2_sumAverage	–0.047
		T2_Elongation	–0.198
	FLAIR	FLAIR_Elongation	0.001
		FLAIR_IntensityVariability	0.256

*FLAIR, fluid-attenuated inversion recovery.*

### Validation of Radiomics Models

**FIGURE 4:**
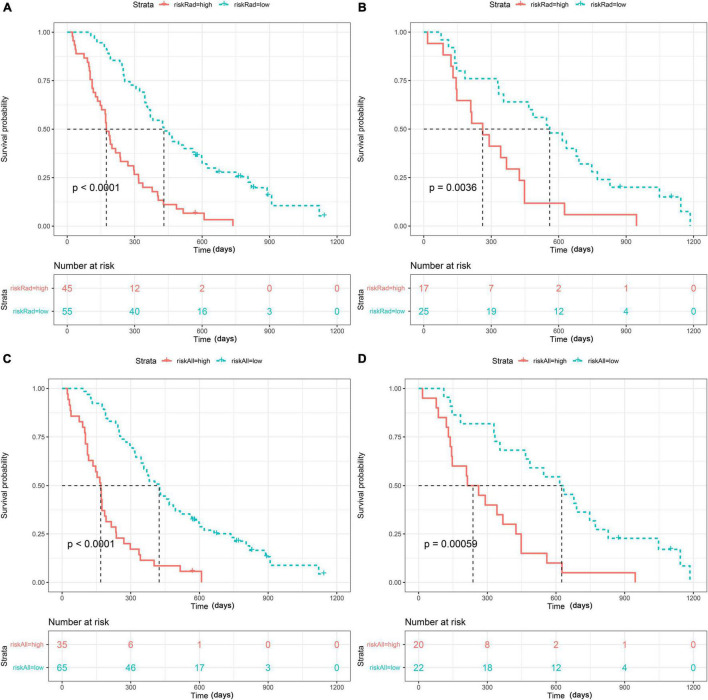
Strata: index for risk stratification. Kaplan–Meier analysis of the overall survival (OS) of patients based on the radiomics model with optimal cutoff values (1.2311) in the training set **(A)** and the test set **(B)** and based on the Radiomics + Clinical model with optimal cutoff values (1.1489) in the training set **(C)** and the test set **(D)**.

As presented in Figure 4 (*p* < 0.001 in the training set and *p* < 0.05 in the test set), both radiomics and Radiomics + Clinical models could effectively stratify the risk of OS. The C-index score in the range 0.74–0.86 (Table 4) in both the training and test sets meant good consistency of the model prediction. In addition, the Radiomics + Clinical model showed better predictive performance than the radiomics model, but the difference was not statistically significant. The overall Schoenfeld global test of the Radiomics + Clinical model was examined for proportional hazards (PH) assumption, which was met (*p* = 0.518). All covariates met the proportional hazards assumption (Supplementary Figure 2). To further evaluate the real performance of the model, we supplemented the 10 times tenfold cross-validation method to randomly divide the dataset 10 times, aiming to perform a more balanced model evaluation by calculating the average and standard deviation values of the model C-index. The C-index scores are shown in Supplementary Table 2.

**TABLE 4:** Comparison of the predictive performance of the models using the C-index in the training and test sets.

Model	C-index (95%CI)	
	Training set	Test set
Radiomics	0.803 (0.744–0.861)	0.764 (0.680–0.848)
Radiomics + Clinical	0.836 (0.785–0.886)	0.799 (0.720–0.878)

### Radiomics Nomogram

**FIGURE 5:**
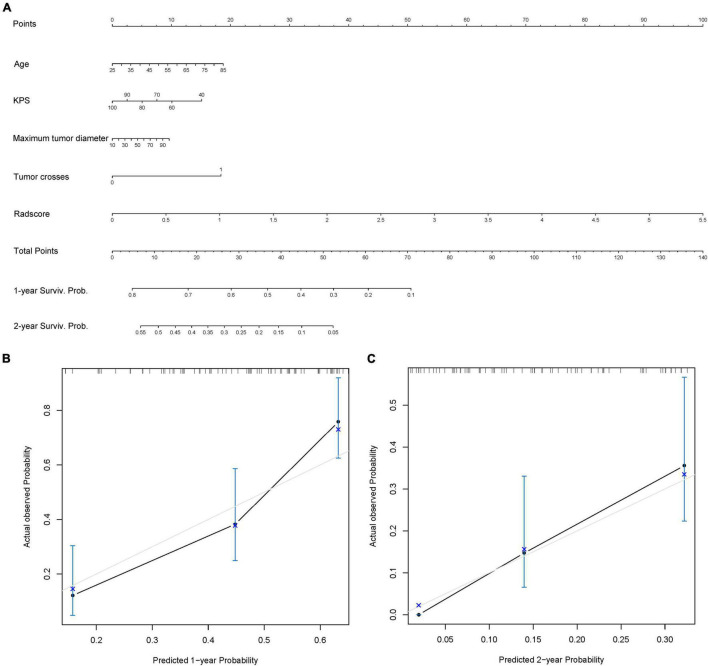
Radiomics nomogram. **(A)** Radiomics + Clinical nomogram constructed by integrating the independent risk factors, including age, Karnofsky performance status (KPS), maximum tumor diameter, tumor crossing the midline, and radscore to predict the 1- and 2-year survival of patients with isocitrate dehydrogenase (IDH)-wild-type glioblastoma (GBM). **(B,C)** Calibration curves used to assess the prediction consistency of the nomogram in the training set **(B)** and the test set **(C)**.

Ultimately, the radiomics signature and four clinical risk factors (age, KPS, tumor crossing the midline, and maximum tumor diameter) were included in the nomogram (Figure 5A). The total score obtained by integrating the individual scores for age, KPS, tumor crossing the midline, maximum tumor diameter, and the radscore can be used to quantitatively predict the probability of a patient’s 1- and 2-year survival (Figure 5A). The calibration curves of OS prediction at 1 and 2 years (Figures 5B,C) showed good agreement between the nomogram prediction and the actual observations.

## Discussion

In this study, we extracted more comprehensive features from CE-T1WI, T2WI, and T2-FLAIR sequences than those in previous studies so that we could mine radiological images in more depth. The radiomics signature has been identified as an independent prognostic biomarker of IDH-wild-type GBM. Therefore, we combined it with clinical risk factors (age, KPS, tumor crossing the midline, and maximum tumor diameter) to construct a combined model and nomogram as a personalized tool to estimate the OS of IDH-wild-type GBM patients. The models were constructed based on a multicenter training queue and verified based on a multicenter test set.

Traditional central nervous system tumor grading is mainly based on histological features, but now, some molecular markers can provide strong prognostic information. Therefore, molecular markers have been added as grading biomarkers and used to further evaluate the prognosis of several tumor types. The 2021 classification criteria of the WHO classified common adult diffuse gliomas into three types: astrocytoma, IDH-mutant; oligodendroglioma, IDH-mutant and 1p/19q-codeleted; and glioblastoma, IDH-wild type. In addition, it should be noted that IDH-wild-type diffuse astrocytic tumors in adults, if microvascular proliferation or necrosis or TERT promoter mutation or EGFR gene amplification or +7/−10 chromosome copy number changes are present, should be diagnosed as IDH-wild-type glioblastoma (Louis et al., 2017, 2021; Brat et al., 2018). Previous studies have explored the prognosis of glioblastoma according to the old classification (Alexander and Cloughesy, 2017; Chaddad et al., 2018; Rao et al., 2018; Zhang et al., 2019). Therefore, on this basis, according to the latest guidelines, we included some tumors previously considered to be of lower grade but now diagnosed with GBM into the study. This updates the prognosis prediction for such tumor patients and provides more real-time help with personalized treatment. This is a more complete and comprehensive retrospective study for patients with newly diagnosed IDH-wild-type GBM. Radiomics uses advanced computing methods to extract potential radiomics features from images and obtain more tumor information in a non-invasive manner. Previous studies have reported that radiomics features can predict the prognosis of gliomas. Bae et al. (2018) showed that radiomic MRI phenotyping can improve survival prediction when integrated with clinical and genetic profiles and thus has potential as a practical imaging biomarker. Tan et al. (2019) showed that radiomics can accurately reflect the heterogeneity of the tumor and edema areas of high-grade glioma so as to reflect the prognostic information to a certain extent. Compared with some previous studies, our model showed similar or even better predictive ability. The C-index of a model constructed for high-grade glioma was 0.764 (Tan et al., 2019). This may be related to the fact that we extracted more features from three different MRI sequences. The combination of multiple sources of information allows for an overall analysis of the tumor and better prognosis prediction. Except as mentioned above, it is more reasonable that multicenter data are adopted in the training and test processes. Although the performance of the constructed models is not the best among existing studies, they have stronger generalization ability, reproducibility, and usability. In addition, to further evaluate the real performance of the model, we supplemented the 10 times tenfold cross-validation method to randomly divide the data set 10 times, aiming to perform a more balanced model evaluation by calculating the average and standard deviation values of the model C-index.

We finally screened out 8 features, including 1 CE-T1WI feature, 5 T2WI features, and 2 T2-FLAIR features. The CE-T1WI sequence can well display the activity and necrotic area of the tumor, the T2WI sequence can reflect the anatomical location and cellularity of the tumor, and the T2-FLAIR sequence can depict the cellularity of the tumor and the density of tumor cells (Tan et al., 2019; Tian et al., 2020). The radiomics features of the combined sequences can represent the characteristics of tumors from different aspects and reflect the information of all aspects of tumors, evaluating tumors and their heterogeneity more comprehensively.

According to the latest classification, we excluded the IDH status and identified the constructed radiomics signature, age, KPS, tumor crossing the midline, and maximum tumor diameter as independent risk factors using multivariable Cox regression analysis. Both the radiomics and Radiomics + Clinical models could effectively stratify the risk of OS in IDH-wild-type GBM. The combined model showed better prediction effect than the single radiomics model, but the effect was limited. This indicates that radiomics features may play a greater role in prognosis prediction. Moreover, it may be partly due to the IDH mutation status factor being reduced by including all IDH-wild-type patients according to the new classification, which may have reflected the importance of IDH mutation status in the prognosis of GBM patients. In addition, the nomogram built based on radiomics features and selected clinical features is a graphical representation of a statistical model and makes the results of the prediction model more prominent and of higher use value. By combining the scores of each risk factor in the nomogram, the 1- and 2-year survival probability of each patient can be estimated, making it a useful tool for the personalized diagnosis and treatment of IDH-wild-type GBM.

However, there are still some limitations in our study. Firstly, manual segmentation will produce personal errors. Automatic tumor segmentation based on deep learning will minimize user bias and facilitate large-scale research. Secondly, due to the heterogeneity of the imaging parameters in the TCGA/TCIA databases, we only included conventional sequences, but did not analyze advanced MRI sequences. Finally, we did not consider the impact of various treatments on tumor progression after standard chemoradiotherapy in the analysis of OS.

## Conclusion

In conclusion, for patients newly classified as IDH-wild-type GBM, radiomics features are still independent prognostic biomarkers, and age, KPS, tumor crossing the midline, and maximum tumor diameter can, to a certain degree, supplement radiomics features. The nomogram constructed by combining radiomics features and relevant clinical factors can improve the individualized prediction of survival of IDH-wild-type GBM patients, which provides help with personalized clinical treatment.

## Data Availability Statement

The original contributions presented in the study are included in the article/Supplementary Material, further inquiries can be directed to the corresponding authors.

## Ethics Statement

The studies involving human participants were reviewed and approved by the Zhongnan Hospital, Wuhan, China (approval No.2020109). The patients/participants provided their written informed consent to participate in this study. Written informed consent was obtained from the individual(s) for the publication of any potentially identifiable images or data included in this article.

## Author Contributions

SW and FX performed the literature search and wrote the manuscript. WS, CY, CM, and YH helped with follow-up. DX, LL, and JC collected the data. HL and HX designed the manuscript structure and wrote the manuscript. All authors read and approved the final manuscript.

## Conflict of Interest

JC was employed by the company GE Healthcare, Shanghai, China. The remaining authors declare that the research was conducted in the absence of any commercial or financial relationships that could be construed as a potential conflict of interest.

## Publisher’s Note

All claims expressed in this article are solely those of the authors and do not necessarily represent those of their affiliated organizations, or those of the publisher, the editors and the reviewers. Any product that may be evaluated in this article, or claim that may be made by its manufacturer, is not guaranteed or endorsed by the publisher.

## Footnotes

1
http://www.Rproject.org
